# Minimally invasive tubular lumbar microdiscectomy in pediatric patients: a single-center case series

**DOI:** 10.1007/s00381-026-07399-2

**Published:** 2026-07-17

**Authors:** Nick De Oliveira, Sam Kavarana, Jim Rogers, Elise Yoon, Stephen Gannon, Christopher M. Bonfield

**Affiliations:** 1 Vanderbilt University School of Medicine, Nashville, TN USA; 2 Division of Neurosurgery, Henry Ford Health Providence Hospital, College of Human Medicine, Michigan State University, Southfield, MI USA; 3 Department of Neurological Surgery, Division of Pediatric Neurological Surgery, Vanderbilt University Medical Center, Doctors Office Tower, 2200 Children’s Way, Suite 9226, Nashville, TN 37232 USA

**Keywords:** Disc herniation, Reherniation, Microdiscectomy, Adolescent spine surgery, Congenital spinal stenosis

## Abstract

**Purpose:**

Minimally invasive tubular lumbar microdiscectomy (MIS-MCD) is used for lumbar disc herniation (LDH) in adults, but pediatric data remain limited. This study aimed to evaluate clinical presentation, perioperative outcomes, and recovery patterns after MIS-MCD, and to explore risk factors for reherniation and reoperation in a pediatric cohort.

**Methods:**

We performed a retrospective case series of consecutive pediatric patients undergoing primary single-level MIS-MCD for LDH from 2014–2024 at a tertiary center by a single surgeon. Patients with primarily traumatic, neoplastic, or infectious pathology were excluded. Primary outcomes included early symptom improvement, return to activities, reherniation, and reoperation. Risk factors for reherniation were explored using Fisher's exact tests.

**Results:**

Thirty-four patients were included (mean age 16.7 ± 1.7 years; 55.9% male; mean BMI 30.6 ± 7.4 kg/m2). Most participated in organized sports (58.9%), with sport/exertional mechanisms being the most common identifiable cause (41.2%). Congenital spinal stenosis (CSS) was present in 20.6%. Mean symptom-to-surgery interval was 9.2 ± 8.3 months. Length of stay was 0.5 ± 0.6 days, median blood loss was 15 mL, and no durotomies occurred. Initial symptom improvement was reported by 94.1%, and 79.4% returned to prior activities. Over a median follow-up of 20.4 months, reherniation occurred in 8 patients (23.5%) and reoperation in 6 (17.6%). Reherniation was significantly associated with CSS (62.5% vs. 7.7%; *p* < 0.001). All reoperations were repeat MIS-MCD; none required fusion.

**Conclusion:**

MIS-MCD is safe and effective in pediatric patients. Reherniation is concentrated in patients with CSS, suggesting preoperative canal narrowing identification may guide counseling and surveillance.

## Introduction

Symptomatic lumbar disc herniation (LDH) is a major cause of pain and disability, and a subset of patients with refractory symptoms ultimately require operative intervention [1, 2]. Microdiscectomy has long been the gold standard for surgical treatment of LDH, specifically the open microdiscectomy (O-MCD) [3–5]. While postoperative outcomes of this approach tend to be favorable, open microdiscectomy necessitates subperiosteal muscle stripping and wider soft-tissue dissection, which may contribute to postoperative pain, prolonged recovery, and paraspinal muscle atrophy [6–8]. To address these limitations, minimally invasive microdiscectomy (MIS-MCD) techniques using tubular retractors were developed to provide a muscle-splitting corridor that preserves posterior musculature while maintaining the principles of microsurgical discectomy [9].

LDH is a much rarer pathology in the pediatric population, and the etiology often includes trauma, developmental abnormalities, or high levels of athletic activity [10, 11]. While O-MCD is proven to be safe and efficacious in children, with large institutional series reporting favorable long-term outcomes, data on MIS-MCD in this population are extremely limited [12, 13]. The earliest report was a 2011 case series of 6 patients, noting the safety and efficacy of the technique. Subsequent case series published in 2018 reported favorable outcomes with low rates of intraoperative blood loss, short hospital stays, and high rates of symptom resolution [14–16]. More recently, a 2021 pediatric series incorporating both O-MCD and MIS-MCD suggested that minimally invasive approaches are feasible and effective in this population [17]. However, the three existing tubular MIS-MCD series in pediatric patients comprise very few patients, and limited analysis has examined reherniation risk factors or long-term reoperation patterns [14, 15, 17].

Despite these encouraging reports, there remain extremely limited data characterizing the clinical presentation, perioperative outcomes, and long-term reoperation patterns in pediatric patients treated with MIS-MCD. In particular, the relative burden of reherniation and the factors associated with recurrence in children are not well defined. Therefore, the objective of this study was to evaluate clinical presentation, perioperative outcomes, and long-term postoperative course after MIS-MCD in a pediatric cohort treated by a single surgeon at a tertiary center with prolonged follow-up. To our knowledge, this series represents one of the largest cohorts of pediatric patients undergoing tubular MIS-MCD and provides insight into the durability and safety of this technique in the pediatric population.

## Methods

### Study design

The present study is a retrospective case series conducted at a single regional tertiary care center with pediatric neurosurgical services. The primary objective was to characterize postoperative outcomes including symptom relief, residual symptoms, healthcare utilization, reherniation, and reoperation after minimally invasive tubular lumbar microdiscectomy (MIS-MCD) in pediatric patients treated by a single surgeon. The study was approved by the Institutional Review Board (IRB#200719), and the requirement for informed consent was waived due to the retrospective nature of the review.

### Operative technique

In all cases, a laminotomy was performed through the tubular retractor to access the herniated disc; no patient underwent an isolated discectomy without bony decompression. In patients with a congenitally small spinal canal, or when there was concern for ongoing compression arising from the lateral recess, facet, or canal itself, the laminotomy was extended to achieve adequate decompression of the lateral dura, the shoulder of the nerve root, and the root as it traversed around the pedicle. A 20-mm tubular retractor was used, which afforded sufficient visualization and working corridor to accomplish this decompression, including in patients with congenital spinal stenosis.

### Cohort selection

Eligible patients were identified using procedural codes and operative logs performed by a single spine and pediatric fellowship-trained neurosurgeon between 2014 and 2024. All consecutive patients treated within the senior author’s pediatric neurosurgical practice were eligible for inclusion. Inclusion criteria were: 1) lumbar disc herniation confirmed by MRI, 2) treatment with a primary single-level MIS-MCD, and 3) at least one documented postoperative neurosurgical follow-up visit. Follow-up duration was calculated from the date of the index MIS-MCD to the last in-system clinical encounter with the Spine team. Follow-up encounters and subsequent operations were captured from the institutional electronic medical record; care received entirely at outside institutions was not systematically captured.

Patients were excluded if they had undergone a concurrent fusion at the index operation or if the procedure was performed primarily for severe traumatic, neoplastic, or infectious pathology. Patients with incomplete medical records preventing reliable extraction of key variables were also excluded. As the database was operation-level, we constructed a patient-level index cohort by identifying each patient’s first MIS-MCD during the study period. Demographics and preoperative characteristics were summarized for this index surgery. All outcomes were analyzed at the patient level; subsequent lumbar operations were used only to determine whether and when a reoperation occurred and did not contribute additional index cases.

### Data collection and outcome measures

Data abstraction was conducted by reviewers using a standardized chart review template. Demographic and clinical variables collected included age, sex, body mass index (BMI), race/ethnicity, athletic status, and comorbidities. Preoperative clinical data included symptomatology (e.g., radicular pain, axial back pain, weakness, numbness, tingling), symptom laterality, mechanism of injury, and time from symptom onset to surgery. Mechanisms of injury were categorized as sport/exertional (onset during athletic, recreational, or physical training activities without a major traumatic event), acute traumatic (onset after a discrete fall, collision, or lifting/twisting injury), insidious/degenerative (gradual onset over weeks to months without a specific precipitating event), or unknown/other (cases lacking a clearly defined injury mechanism or timeline). Comorbidities included diabetes mellitus, hypertension, bone pathology (osteoporosis, osteoarthritis, or inflammatory arthritis), and congenital spinal stenosis (CSS) which was recorded when explicitly described in the clinical radiology report read or in the interpreting spine surgeon’s review at the time of initial consultation. CSS was therefore based on qualitative expert assessment rather than standardized numerical cutoffs.

Operative data included surgical level, laterality of decompression, and presence of intraoperative complications such as dural tears. Herniation location within the canal was classified on preoperative axial MRI as central, paramedian/subarticular, combined central and paramedian, or foraminal/far-lateral, based on the position of the herniated fragment relative to the midline, lateral recess, and pedicle. Postoperative data collected included length of hospital stay, documentation of symptom improvement, presence of residual symptoms, and whether the patient returned to prior activities. Residual symptoms at early follow-up were defined as back or leg symptoms that were clearly improved compared with baseline but not fully resolved, irrespective of intensity. In practice, these were typically documented as mild, subjectively improved symptoms that did not clearly limit daily activities. Return to prior activities was abstracted from clinic documentation and encompassed return to school, usual recreational activity, or activities of daily living at or near the preoperative level. Early outcomes were defined as initial symptom improvement, residual symptoms, and return to prior activities at approximately the 3-month postoperative visit.

### Outcomes

Outcomes of interest included postoperative symptoms and functional status (e.g., return to activities, residual symptoms), reherniation, and reoperation at the same or adjacent level with time to reoperation recorded, when applicable. Reherniation was defined as recurrent radicular symptoms and confirmed reherniation of the disc at the index or a new lumbar level on imaging. Recurrences were categorized as same level/side, same level/contralateral side, or different level. Reoperation was defined as any subsequent lumbar spine operation after the index MIS-MCD during the study period, including repeat microdiscectomy and more extensive decompression and/or fusion procedures, regardless of level or indication. Secondary outcomes included rates of emergency department (ED) visits within 3 months postoperatively and readmission rates within 6 months postoperatively.

### Statistical analysis

Continuous variables such as age at surgery, BMI, time from symptom onset to surgery, length of stay, and length of follow-up were summarized as mean with standard deviation or as median with interquartile range according to distribution. Categorical variables such as sex, race/ethnicity, mechanism of injury, and initial symptom improvement were summarized as counts and percentages. To explore potential risk factors for reherniation, patients with and without reherniation were compared using Student’s t-test or the Mann–Whitney U test for continuous variables, and Pearson’s chi-square test or Fisher’s exact test for categorical variables when expected cell counts were small. Descriptive statistics and all outcome comparisons were patient-level and anchored to the index surgery. A two-sided p value < 0.05 was considered statistically significant. All analyses were performed in SPSS Version 29 (IBM, Armonk, NY).

## Results

### Patient demographics and preoperative characteristics

A total of 34 pediatric patients underwent primary MIS-MCD for LDH during the study period (Table 1). Mean age at surgery was 16.7 ± 1.7 years (range 13.6–21.8), and 55.9% were male. Most patients identified as White (73.5%), and mean BMI was 30.6 ± 7.4 kg/m2. Comorbidities were uncommon (5.9%), with no patients having diabetes mellitus. Congenital spinal stenosis (CSS) was present in 7 patients (20.6%). The majority of patients (58.9%) were involved in organized sports. Sport/exertional mechanisms were the most common identifiable cause of injury (41.2%), followed by acute traumatic mechanisms (11.8%). At presentation, all patients reported pain, while weakness (11.8%), numbness (11.8%), and tingling (2.9%) were less common. Radicular pain was the primary presenting complaint in all patients who proceeded to surgery; no patient underwent MIS-MCD for isolated axial back pain. Conservative management in the preoperative phase including medications (100%) and physical therapy (97.1%) was nearly universal; epidural steroid injections were performed in 29.4% of patients.

**Table 1:** Patient demographics and preoperative characteristics

Variable	Pediatric (*n* = 34)
Age at Surgery (years)	16.7 ± 1.7
Gender	
Male	19 (55.9%)
Female	15 (44.1%)
Race	
White	25 (73.5%)
Non-White	9 (26.5%)
BMI (kg/m^2^)	30.6 ± 7.4
Any Comorbidity	2 (5.9%)
Diabetes mellitus	0 (0.0%)
Hypertension	1 (2.9%)
Bone pathology	1 (2.9%)
CSS	7 (20.6%)
Organized Sport Participation	20 (58.9%)
Insurance	
Private	21 (61.8%)
Public	7 (20.6%)
Military	2 (5.9%)
Uninsured	4 (11.8%)
Mechanism of Injury	
Sport/exertional	14 (41.2%)
Acute traumatic	4 (11.8%)
Insidious/degenerative	0 (0.0%)
Other/unknown	16 (47.1%)
Patient Symptoms*	
Radicular Pain	34 (100.0%)
Weakness	4 (11.8%)
Numbness	4 (11.8%)
Tingling	1 (2.9%)
Laterality of Symptoms	
Left	14 (41.2%)
Right	14 (41.2%)
Both	6 (17.6%)
Conservative Management*	
Medication	34 (100.0%)
Physical Therapy	33 (97.1%)
Epidural Steroid Injection	10 (29.4%)

^*^Patients may have more than one symptom and may have received more than one form of conservative management. *CSS* congenital spinal stenosis. Bone pathology includes osteoporosis, osteoarthritis, or inflammatory arthritis

### Operative course and surgical details

**Fig. 1:**
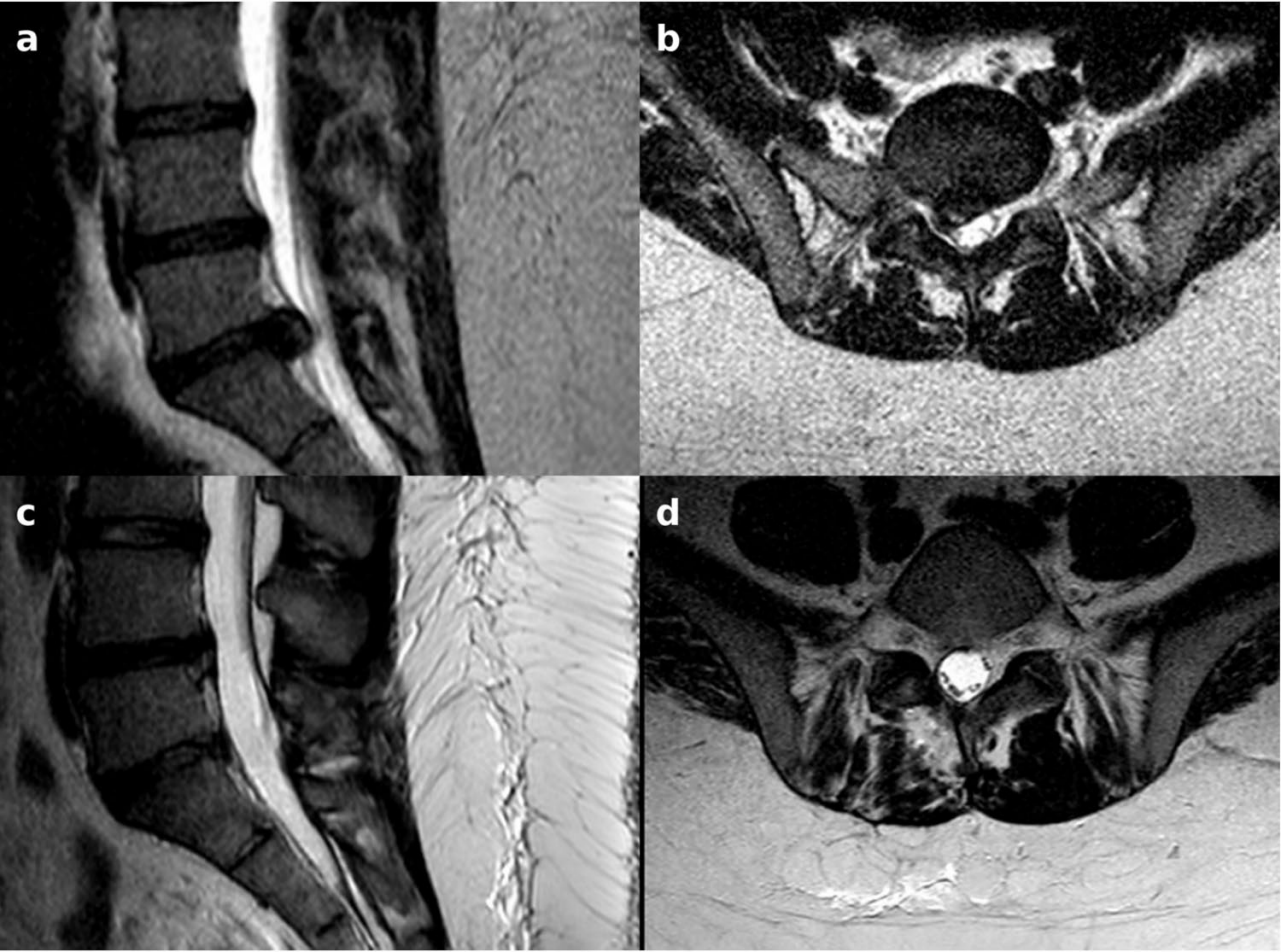
Pre- and postoperative MRI of a 16-year-old female with a right L5–S1 lumbar disc herniation treated by right L5–S1 minimally invasive microdiscectomy (MIS-MCD). (**a**) Preoperative T2-weighted sagittal MRI; (**b**) preoperative T2-weighted axial MRI; (**c**) postoperative T2-weighted sagittal MRI; (**d**) postoperative T2-weighted axial MRI

Operative details are summarized in Table 2. Mean time from symptom onset to surgery was 9.2 ± 8.3 months. L5-S1 was the most treated level (58.8%), followed by L4-5 (38.2%). Surgical decompression was right-sided in 55.9% of cases. On preoperative MRI, herniations were paramedian in 19 patients (55.9%) and central in 12 (35.3%), with 3 patients (8.8%) demonstrating a combined central and paramedian herniation. No herniation was foraminal or far-lateral. Estimated blood loss was low (median 15 mL [IQR 5–50]). There were no intraoperative durotomies. Hospital length of stay was short (0.5 ± 0.6 days). A representative case is shown in Fig. 1.

**Table 2:** Operative course

Variable	Pediatric (*n* = 34)
Time from Symptom Onset to Surgery (months)	9.2 ± 8.3
Operative level	
L1–2	0 (0.0%)
L2–3	0 (0.0%)
L3–4	1 (2.9%)
L4–5	13 (38.2%)
L5–S1	20 (58.8%)
Laterality of Surgery	
Left	15 (44.1%)
Right	19 (55.9%)
Herniated Disc Location in the Spinal Canal	
Paramedian	19 (55.9%)
Central	12 (35.3%)
Central and Paramedian	3 (8.8%)
Foraminal/Far-lateral	0 (0.0%)
Intraoperative Durotomy	0 (0.0%)
Estimated Blood Loss (mL), median [IQR]	15 [5–50]
Length of Hospital Stay (days)	0.5 ± 0.6

Continuous variables are presented as mean ± standard deviation unless otherwise indicated. IQR = interquartile range

### Postoperative outcomes and complications

Early postoperative outcomes and complications are presented in Table 3. Initial symptom improvement was reported by 94.1% of patients, and 79.4% returned to their prior activities. Residual symptoms at early follow-up were documented in 47.1% of patients. Early postoperative healthcare utilization was low: one patient (2.9%) had an emergency department visit within 3 months, and two patients (5.9%) were readmitted within 6 months. One patient (2.9%) developed a deep surgical site infection requiring operative debridement. Total clinical follow-up duration was variable, with an average of 32.8 ± 31.4 months (median 20.4 months, IQR 4.7–53.7).

**Table 3:** Postoperative outcomes and complications

Variable	Pediatric (*n* = 34)
Early Postoperative Outcomes	
Initial symptom improvement	32 (94.1%)
Return to prior activities	27 (79.4%)
Residual symptoms	16 (47.1%)
Healthcare Utilization	
ED visit within 3 months	1 (2.9%)
Readmission within 6 months	2 (5.9%)
Complications	
Deep surgical site infection	1 (2.9%)
Reherniation	8 (23.5%)
Same level, same side	6 (75.0%)
Same level, contralateral side	1 (12.5%)
Different level	1 (12.5%)
Reoperation	6 (17.6%)
Repeat MIS-MCD	6 (100.0%)
Fusion or laminectomy	0 (0.0%)
Time between surgeries (months), median [IQR]	9.3 [4.6–59.1]
Length of follow-up (months), median [IQR]	20.4 [4.7–53.7]

Percentages for reherniation subtypes are calculated within the reherniation group (*n* = 8); percentages for reoperation subtypes are calculated within the reoperation group (*n* = 6). *ED* emergency department, *IQR* interquartile range, *MIS-MCD* minimally invasive tubular lumbar microdiscectomy

### Reherniation and reoperation analysis

**Fig. 2:**
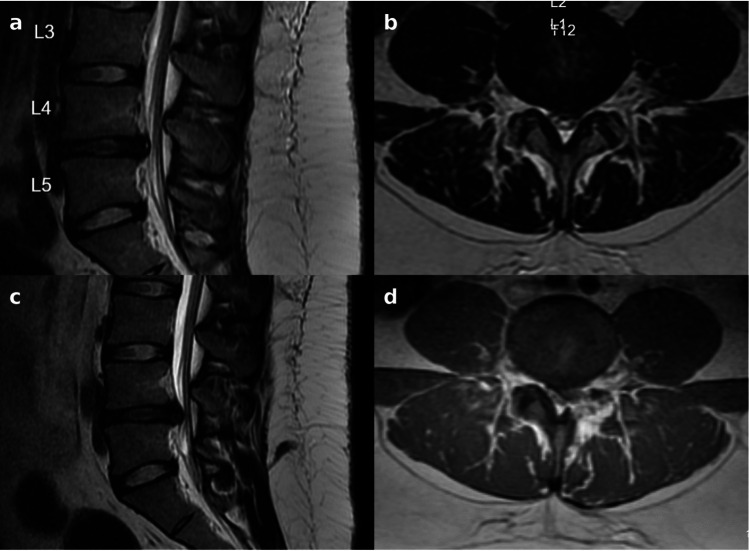
Pre- and postoperative MRI of an 18-year-old female with a left L4–5 lumbar disc herniation and CSS treated by left L4–5 minimally invasive microdiscectomy (MIS-MCD), with recurrent left L4–5 herniation three years after the index procedure. (**a**) Preoperative T2-weighted sagittal MRI; (**b**) preoperative T2-weighted axial MRI; (**c**) T2-weighted sagittal MRI at recurrence; (**d**) T2-weighted axial MRI at recurrence

Reherniation occurred in 8 of 34 patients (23.5%), and reoperation was required in 6 patients (17.6%) (Table 3). Among reherniations, the majority were at the same level and same side (75.0%), with one at the same level/contralateral side (12.5%) and one at a different level (12.5%). All reoperations were repeat MIS-MCD; no patient required laminectomy or fusion. Median time from index surgery to reoperation was 9.3 months (IQR 4.6–59.1). To explore potential risk factors for reherniation, we compared characteristics of patients with and without reherniation (Table 4). CSS was the only variable with a significant association with reherniation (5/8 [62.5%] vs. 2/26 [7.7%]; Fisher’s exact p < 0.001). Other baseline features including age, sex, BMI, organized sports participation, presenting symptoms, mechanism of injury, and time from symptom onset to surgery did not differ between groups. A representative reherniation case in a patient with CSS is shown in Fig. 2.

**Table 4:** Patient characteristics by reherniation status

Variable	Reherniation (*n* = 8)	No reherniation (*n* = 26)	*p*-value
Age at Surgery (years)	16.7 ± 1.3	16.7 ± 1.9	0.972
Gender			0.417
Male	3 (37.5%)	16 (61.5%)	
Female	5 (62.5%)	10 (38.5%)	
BMI (kg/m2)	33.8 ± 8.2	29.7 ± 7.0	0.182
Organized Sport Participation	5 (62.5%)	15 (57.7%)	1.000
Symptoms*			
Radicular Pain	8 (100.0%)	26 (100.0%)	n/a
Weakness	0 (0.0%)	4 (15.4%)	0.551
Numbness	1 (12.5%)	3 (11.5%)	1.000
Tingling	0 (0.0%)	1 (3.8%)	1.000
Any Comorbidity	1 (12.5%)	1 (3.8%)	0.422
Diabetes mellitus	0 (0.0%)	0 (0.0%)	n/a
Hypertension	1 (12.5%)	0 (0.0%)	0.235
Bone pathology	0 (0.0%)	1 (3.8%)	1.000
Congenital Spinal Stenosis	5 (62.5%)	2 (7.7%)	< 0.001
Mechanism of Injury			0.971
Sport/exertional	3 (37.5%)	11 (42.3%)	
Acute traumatic	1 (12.5%)	3 (11.5%)	
Insidious/degenerative	0 (0.0%)	0 (0.0%)	
Other/unknown	4 (50.0%)	12 (46.2%)	
Time from Symptom Onset to Surgery (months)	11.6 ± 12.0	8.4 ± 7.0	0.360

^*^Patients may present with more than one symptom. *CSS* congenital spinal stenosis. Bone pathology includes osteoporosis, osteoarthritis, or inflammatory arthritis. Continuous variables were compared using Student’s t-test or the Mann–Whitney U test; categorical variables were compared using Pearson’s chi-square or Fisher’s exact test as appropriate. n/a indicate comparisons that could not be computed (both groups at 0% or 100%)

## Discussion

In this single-surgeon, single-center case series of 34 pediatric patients undergoing minimally invasive tubular lumbar microdiscectomy (MIS-MCD), the technique demonstrated high rates of early symptom improvement, short hospital stays, low perioperative morbidity, and low acute-care utilization. Reherniation occurred in approximately one-quarter of patients and was strongly associated with congenital spinal stenosis (CSS). Importantly, all reoperations were successfully managed with repeat MIS-MCD, and no patient required fusion.

### Comparison with existing literature

The pediatric literature on lumbar disc herniation is dominated by open or mixed open/MIS series but consistently demonstrates excellent pain relief, rapid return to activity, and low complication rates [10–14, 18]. Our cohort is consistent with these findings, as most children had symptomatic improvement, hospital stays were typically ≤ 1 day, and most returned to prior activities, supporting MIS-MCD as a safe and effective technique in appropriately selected youth. The low rate of intraoperative complications, including no durotomies, and minimal blood loss (median 15 mL) are also consistent with the favorable safety profile reported in prior MIS-MCD series. Thomas et al. reported a mean estimated blood loss of 10.8 mL with zero complications in 6 patients [15], while Montejo et al. reported 91% excellent or good outcomes by modified Macnab criteria with no intraoperative or postoperative complications in 12 patients [14].

We observed a reherniation rate of 23.5% and a reoperation rate of 17.6%, which are higher than recurrence rates of 5–15% typically reported in the adult literature, though direct pediatric comparisons remain limited [17–21]. This may reflect several factors unique to our cohort, including a high proportion of patients with CSS (20.6%), likely reflecting referral-based selection toward more symptomatic anatomy, a predominance of sport-related mechanisms, relatively long follow-up in a subset of patients, and the small sample size. The strong association between CSS and recurrence in our cohort is consistent with prior work linking adolescent disc herniation and reherniation to developmental canal narrowing and apophyseal variants; prior MRI-based studies have demonstrated that adolescents requiring surgical intervention have significantly smaller spinal canal dimensions than those managed conservatively [17, 22]. Despite this higher recurrence burden, all pediatric failures were managed successfully with repeat MIS microdiscectomy and no child required fusion, in line with prior reports that fusion for isolated pediatric disc herniation is rarely necessary [12, 13]. Two features of our series may further contribute to the observed recurrence rate. First, although all patients were pediatric at the index operation, the senior author continues to follow these patients into adulthood, including after transition to the adult hospital, such that follow-up was not truncated by transfer of care. Our series may therefore capture delayed recurrences that would go undetected in pediatric series in which surveillance ends at the pediatric institution.

Second, we considered whether the tubular corridor itself may have contributed to incomplete decompression. A laminotomy was performed in every case, and in patients with a narrow canal or lateral recess stenosis the laminotomy was extended to decompress the lateral dura and the traversing root around the pedicle; the 20-mm working channel permitted this without conversion to an open exposure, and no patient underwent an isolated discectomy despite known canal narrowing. Notably, no herniation in this cohort was foraminal or far-lateral, the configurations in which the lateral visualization afforded by a tubular corridor is most constrained. Reoperation occurred at a median of 9.3 months rather than in the early postoperative period, a pattern more consistent with true recurrent herniation than with retained or missed fragments. Nonetheless, the tubular approach affords a narrower corridor and more limited lateral visualization than an open exposure, and a contribution of incomplete decompression in individual cases cannot be excluded.

The high proportion of athletes in our cohort (58.9%) warrants comment, as return to sport is a primary concern for adolescent patients and families. Our finding that 79.4% returned to prior activities is broadly consistent with published data in young athletes; Cordover et al. reported that 71% of high school and college-age athletes returned to play at a mean of 4.5 months after lumbar microdiscectomy [23], and Menger et al. reported 82.4% return to sport in their pediatric MIS series [16]. However, return to organized sport timing was not available in our cohort, and future studies should include sport-specific return-to-play outcomes.

The relatively long preoperative symptom duration in our cohort (mean 9.2 months) may reflect delayed recognition of disc herniation in adolescents, more conservative initial management of back pain in younger patients, or longer pathways through primary and sports medicine care before surgical referral [10, 11]. Epidural steroid injections were used in approximately one-third of patients, which is consistent with the generally more conservative approach to pediatric LDH management, although the evidence base for epidural injections in the pediatric population remains limited [10, 11, 24]. The elevated mean BMI in our cohort (30.6 kg/m2) is also notable, as population-based analyses of pediatric lumbar disc herniation have identified obesity as a significant independent risk factor, with surgical patients having a substantially higher mean BMI than matched controls [25]. Lastly, our finding that 47.1% of patients had residual symptoms at early follow-up warrants comment, as it might appear to conflict with the high rate of initial symptom improvement (94.1%). However, our intentionally broad definition captured any persistent back or leg symptoms that had not fully resolved, regardless of severity, and the vast majority of these patients nonetheless returned to prior activities.

### Clinical implications

Pediatric patients and families can be reassured regarding short-term safety and return to prior activities after MIS-MCD [10–13]. However, those with CSS or other structural abnormalities should be counseled about a higher risk of recurrence and followed more closely. Given that reherniation in our cohort clustered in patients with CSS, preoperative identification of congenital canal narrowing may help guide counseling and postoperative surveillance strategies. Importantly, when recurrence does occur, repeat MIS-MCD appears sufficient in pediatric cases without the need for more extensive procedures such as laminectomy or fusion. This finding is clinically reassuring for families and supports a stepwise surgical approach to pediatric disc herniation [13]. Symptom-triggered rather than scheduled surveillance is reasonable given that time to reoperation was variable (median 9.3 months).

### Limitations and future directions

This study has several limitations. It is a retrospective, single-surgeon, single-center series with a modest sample size and few reherniation events, which limits generalizability, reduces statistical power, and introduces potential selection and documentation bias. Because event counts were low, we did not perform multivariable modeling. All reported associations are unadjusted, and the numerous univariate comparisons without correction for multiple testing should be viewed as hypothesis-generating rather than definitive. Importantly, CSS was defined by qualitative expert assessment from radiology reports and the interpreting surgeon’s review; although the single-surgeon design provides consistency in this determination, the lack of quantitative criteria limits reproducibility of this finding. Follow-up duration was heterogeneous and based on documented in-system encounters, so patients who recovered uneventfully and did not return may appear to have short follow-up. This pattern may inflate the observed reherniation rate because patients who reherniated necessarily returned for further care, whereas patients who recovered uneventfully may not have returned; conversely, it may also underestimate recurrences occurring outside our system, despite a substantial subset being followed for 3–5 years or longer. Detailed radiographic variables and standardized patient-reported outcome measures (PROMs) were not routinely collected, limiting detailed functional and radiographic comparison with other series. Outcomes also relied on non-standardized clinical documentation rather than validated PROM instruments. Finally, we did not perform time-to-event analyses because follow-up was variable and not uniformly captured longitudinally, which limits reliable estimation of censoring and event timing. More broadly, no randomized controlled trial has compared MIS-MCD with open microdiscectomy in pediatric patients, and no systematic review has examined reherniation risk factors specific to the pediatric population, which limits the evidence base against which our findings can be interpreted. Our findings are also specific to a tubular microsurgical technique. Full-endoscopic discectomy is not performed at our pediatric institution and is not within the operative practice of the senior author; we are therefore unable to compare tubular MIS-MCD with endoscopic approaches, which merit dedicated study in the pediatric population.

Future work should validate these findings, particularly the association between CSS and reherniation, in larger, multicenter cohorts using standardized PROMs, detailed imaging and intraoperative variables, and time-to-event methods. Prospective registries or pragmatic trials could also test whether tailored return-to-sport protocols for high-level youth athletes after MIS-MCD reduce recurrence and improve long-term quality of life. Additionally, studies incorporating standardized canal dimension measurements rather than qualitative CSS assessment would strengthen the evidence base for this risk factor.

## Conclusion

Minimally invasive tubular lumbar microdiscectomy is safe and effective in pediatric patients, providing high rates of early symptom improvement, short hospitalization, and low perioperative morbidity. Reherniation is concentrated in patients with congenital spinal stenosis and is amenable to repeat MIS-MCD without the need for fusion. These findings support the use of MIS-MCD as a durable technique for pediatric lumbar disc herniation and underscore the importance of preoperative identification of CSS for counseling and follow-up planning.

## Data Availability

No datasets were generated or analysed during the current study.
